# Analysis of differences between total IgG and sum of the IgG subclasses in children with suspected immunodeficiency – indication of determinants

**DOI:** 10.1186/s12865-018-0259-7

**Published:** 2018-06-27

**Authors:** Gerard Pasternak, Aleksandra Lewandowicz-Uszyńska, Katarzyna Pentoś

**Affiliations:** 1 3rd Department and Clinic of Paediatrics, Immunology and Rheumatology of Developmental Age, Wroclaw Medical University, L. Pasteura 1, Wroclaw, 50-367 Poland; 2 Department of Immunology and Paediatrics, Provincial Hospital J. Gromkowski, Koszarowa 5, Wroclaw, 51-149 Poland; 3 Institute of Agricultural Engineering, Wroclaw University of Environmental and Life Sciences, J. Chełmońskiego 37/41, Wroclaw, 51-630 Poland

## Abstract

**Background:**

Deficits in disorders of humoral immunity associated with a deficit of antibodies are the most common primary immunodeficiency. Total IgG and IgG subclasses measurements are used to diagnose, differentiate and control in patients with primary and secondary immunodeficiencies.

**Methods:**

The purpose of the study was to analyze the structure patients group according to difference between total IgG and sum of the IgG subclasses and to determine factors affecting the level of this difference. This study was based on data collected from 670 children referred to the Department of Clinical Immunology and Pediatrics in order to diagnose the immune disorders. For all children the level of the total of immunoglobulins IgG and of the IgG subclasses (IgG1, IgG2, IgG3, IgG4) were determined. The group of children was divided into subgroups according to gender, age (under 6 years of age, 6.5–12 years, and 12–18 years), and IgG abnormality (below the normal range, normal and above the normal range). In the patients group, the total IgG values were on average higher than sum of the IgG subclasses.

**Results:**

Statistical analysis shown the all parameters under study (age, gender and IgG abnormality) influence statistically significant on the discrepancy between the sum of the IgG subclasses and total IgG. Assessment of IgG and IgG subclasses levels is based on different methods what causes the discrepancy between the sum of the IgG subclasses and total IgG.

**Conclusions:**

Standardization in that regard is crucial. In addition, we have shown the reliability of the results obtained. Despite the determination in two different laboratories and on different analyzers, as well as the freezing process does not affect the test results.

## Background

Primary Immunodeficiencies (PID) are a heterogeneous group of diseases. These are rare diseases, but not as rare as one might expect. According to Lim and Elenitoba-Johnson (2004), approximately 400 new PID cases are diagnosed annually in the United States [1]. The course of these diseases can be extremely severe and the high risk other of infections can lead to life-threatening conditions. Delays in making the right diagnosis often results in extremely severe clinical conditions and numerous complications. The speed and accuracy of diagnosis, as well as the implementation of appropriate treatment, will lead to significant benefits for the health and life of the patient.

Most common among PIDs are disorders of the humoral response associated with deficiency of antibodies, a heterogeneous group of proteins present in the body fluids of all vertebrates and characterized by a common pattern of construction. The most important element of the humoral response in the human body are immunoglobulins and the most important of these are immunoglobulins of the IgG class which account for about 75% of all immunoglobulins contained in serums. Within the variable parts of immunoglobulins, there are subtle structural differences that determine antigenic properties and affect effector functions associated with, among other things, complement activation and binding to one or more Fc antibody receptors present among others present on phagocytic cells. Because of the molecular structure, 4 subclasses of this immunoglobulin – IgG1,2,3 and 4 are distinguished. Irregularities in the concentrations of particular IgG subclasses may condition various disease states. The most common IgG1 deficiency is the result of a generalized deficiency of antibodies [2]. IgG2 deficiency is associated with recurrent viral and bacterial infections [3]. IgG3 deficiency is observed in viral infections of the urinary tract; IgG2 and IgG3 deficiency predisposes to recurrent respiratory tract infections [4]. IgG4 deficiency is diagnosed in chronic bronchial and lung diseases [5].

For the correct interpretation of the concentrations of immunoglobulins IgG and IgG subclasses in serums, it is important to know the reference ranges of concentrations of immunoglobulins, depending on the age of the patient. The production of immunoglobulins changes during the natural maturation of the immune system in healthy populations. Immunoglobulin production begins during fetal stage. Up to 6 months of age, the baby’s blood circulates immunoglobulins obtained through the placenta from the mother. During this period, their own immunoglobulin production gradually increases in response to stimulation with food antigens and microorganisms. From 6 months of age, intensive individual antibody production begins. Causes of deficiency in the production of immunoglobulins, the so-called hypogammaglobulinemia, include primary and secondary immunodeficiency.

The essence of carrying out a reliable diagnosis of PID is largely dependent on an accurate medical history. The data obtained from an interview will determine the direction of further investigation, mainly laboratory based testing. In our department, we routinely evaluate IgG in every patient with suspected PID. In specific cases, we evaluate the IgG subclasses. Samples are usually taken one day apart (total IgG and IgG subclasses).

To the best of the authors’ knowledge, there is a lack of analysis concerning differences between total IgG and sum of the IgG subclasses in children (by age group and sex) with suspected immunodeficiency, according to the difference of IgGsum and IgG [5–8].This study aimed to assess frequency and degree of such discrepancies in routine samples. Data was collected retrospectively from 670 children (aged 2 months to 18 years) referred to the Department of Clinical Immunology and Paediatrics for the purpose of diagnosis of immune disorders. All children were screened, among other things, for the total of immunoglobulins IgG and of the IgG subclasses (IgG1, IgG2, IgG3, IgG4). The group of patients was divided into subgroups according to age (under 6 years of age, in the age range between 6 and 12 years, and in the age range between 12 and 18 years), gender, and IgG abnormality (below the normal range, normal and above the normal range).

## Methods

Each year in the Department of Immunology and Paediatrics, we treat about 1300 children (aged 6 months to 18 years). These are patients referred to us for diagnostic purposes of primary immunodeficiencies. Most of them are children aged 0–6 years and the majority of these are children with recurrent respiratory tract infections. In our geographic area (Central and Eastern Europe), 6–8 fever infections per year we treat as a norm. All children were screened routinely, for among other things, the total of the IgG in serum. Using our own experience as well as Jeffrey Modell Foundation criteria, in some cases we mark IgG subclasses (IgG1, IgG2, IgG3, IgG4) depending on the individual indications for the patient. Mostly, economic conditions cause us to freeze samples and send them to another lab. The most common sample for IgG subclasses is taken the next day from the sample of total IgG.

The ARTITECT cSystem by immunoturbidimetric method is used for total IgG determination. In contrast, IgG subclass levels are measured by the nephelometric method with the use of a BN ProSpec Siemens analyzer. Both analyzers use a set of reagents provided by the manufacturers.

Statistical analysis was based on the t-test, Kruskal–Wallis test and Mann-Whitney U test, with a significance level of 5% (*p* ≤ 0.05). The data analyses were carried out using Statistica version 10 software.

All procedures performed in studies involving human participants were ‘in accordance’ with the ethical standards in compliance with the Helsinki Declaration. Bioethical committee of Wroclaw Medical University approval number 638/2017.

## Results and Discussion

Data from medical tests of 670 children aged between 2 months and 18 years were used in this research. The median age of patients was 4.2 years with 421 males (62,8%) and 249 females (37,2%). The summary statistics for each analysis is detailed in Table 1.

**Table 1:** Mean, median, and standard deviation for IgG, IgGsum, and IgG subclasses

Subclasses	Median [g/l]	Mean [g/l]	Standard deviation [g/l]
IgG	7,33	7,79	3,38
IgGsum	6,96	7,36	3,12
IgG1	4,76	5,05	2,16
IgG2	1,34	1,57	1,02
IgG3	0,26	0,31	0,23
IgG4	0,17	0,42	0,69

For each patient, parameter D proposed by authors (discrepancy between IgGsum and IgG expressed as a percentage of IgGsum) was calculated as follows:

$$ D=\frac{IgGsum- IgG}{IgGsum}\cdot 100\% $$

where IgGsum is the sum of subclasses (IgG1+ IgG2+ IgG3+ IgG4), IgG is the total of the IgG in serum.

In the group of 670 children, there were 22 patients with D parameter exceeding 40% who were excluded from further analysis.In the course of the investigation, it was demonstrated that these were samples (IgG total and IgG subclasses) obtained at intervals greater than one day.

In the group of 648 children considered for further analysis, 245 (37.8%) were female and 403 (62.2%) were male; 428 were under 6 years of age, 151 were in the age range between 6 and 12 years and 69 were in the age range between 12 and 18 years (66.0, 23.3 and 10.7%, respectively); for 80 patients IgG was below the normal range, for 496 patients IgG was normal, and for 73 patients IgG was above the normal range (12.3, 76.5 and 11.2%, respectively).The group of 80 patients with Total IgG deficiency can be divided according to the medical history into three clinical subgroups: recurrent respiratory tract infections (RRT), primary immunodeficiency (PID) and transient hypogammaglobulinemia of infancy (THI).

In this group 55 patients had RRT, 20 PIDs and 5 THI. In PIDs group was: 1 patient with Netherton syndrome, 1 patient with complement deficiency, 1 patient with Nijmegen breakage syndrome, 1 patient with ELANE-related neutropenia, 1 patient with common variable immunodeficiency (CVID), 1 patient with IgA deficiency, 3 patients with ataxia-telangiectasia syndrome, 11 patients with phagocytic disorders.

**Fig. 1:**
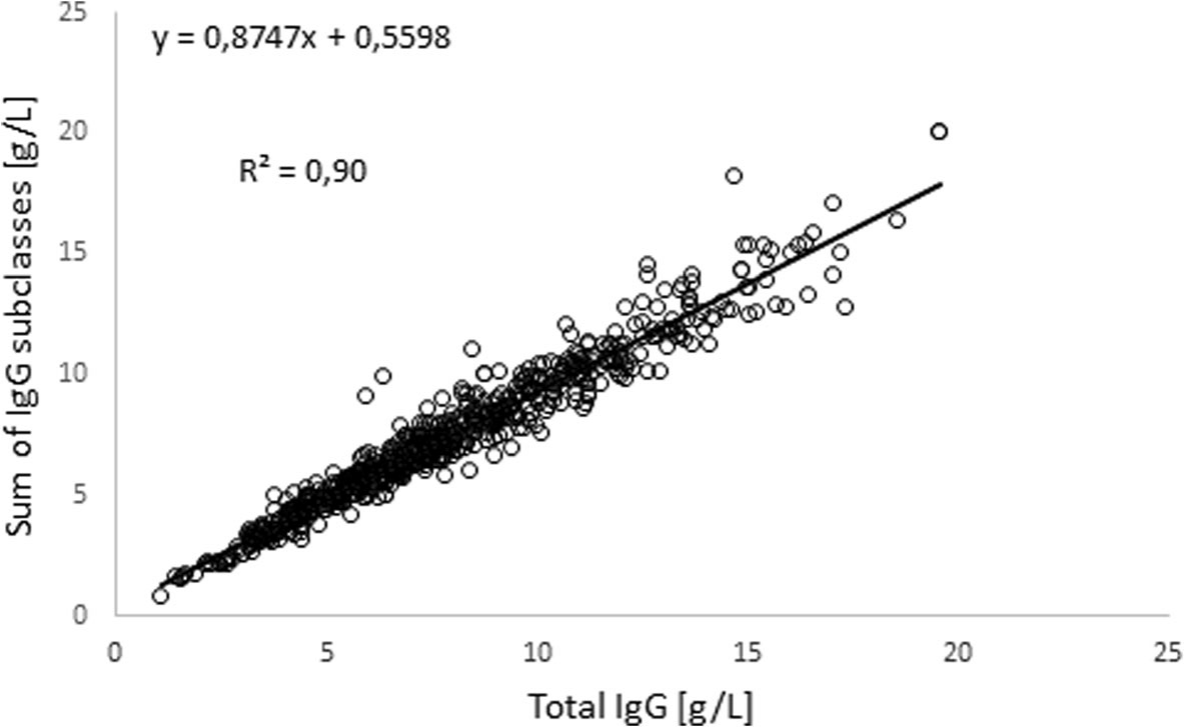
Scatter plot of IgG versus sum of IgG subclasses

Significant correlation was observed between IgG and IgGsum as shown in Fig. 1.

Similar results were presented by McLean-Tooke et al. (2013) for 571 patients aged 0.5–83.6 years [9].

The D parameter was used for dividing the population into subgroups: D < 5% (0–5%); D < 10% (0–10%); D < 15% (0–15%); D > 15% (15–40%). Results of IgG were on average 5.8% higher than IgGsum. The results of statistical analysis (t-test) show that there is a statistically significant difference (*p* < 0.05) between total IgG and IgGsum. Of 648 samples, 102 (15.7%) had D value > 15%. In this group, 95 (93%) had an IgG above IgGsum. In the case of the other 546 patients, 416 (64.2% of the whole population) had a difference of IgGsum from IgG < 10% and 238 (36.7% of the whole population) had this difference < 5%. In the group with D value < 10%, 272 (65.4%) had an IgG above IgGsum. In the case of the group with D value < 5%, 137 patients (57,5%) had an IgG higher than IgGsum. Comparing these results with those presented by McLean-Tooke et al. (2013) (IgGsum were on average 3.9% higher than IgG) can lead to the conclusion that the child population is different from adults according to the discrepancy between IgG and IgGsum.

Statistical analysis based on Pearson Correlation Coefficientshowed no statistically significant correlation (*p* < 0.05) between D value and IgG1 (*r* = 0.016), IgG2 (*r* = − 0,049), IgG3 (r = − 0,035) or IgG4 (r = 0,002).Based on the Kruskal–Wallis test results, statistically significant differences were identified between the D value affected by age (*p* = 0.022), gender (*p* = 0.003), and IgG abnormality (*p* = 0.000).

**Fig. 2:**
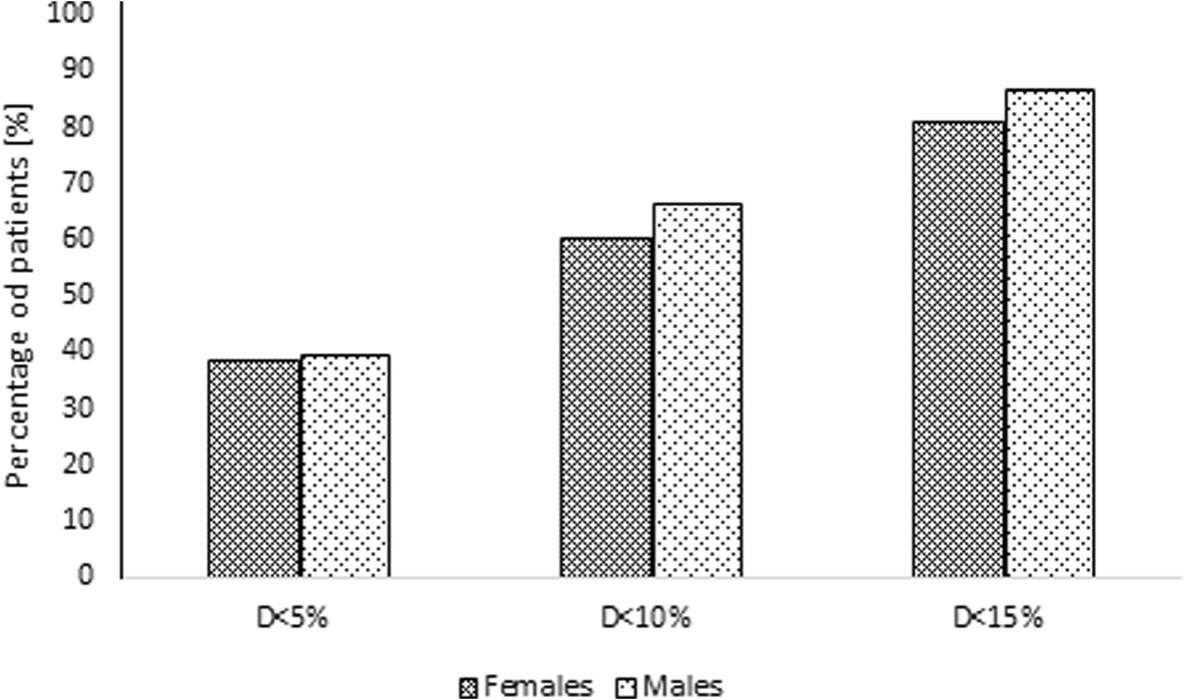
Percentage of males and females according to the difference of IgGsum and IgG for the whole patient population

**Fig. 3:**
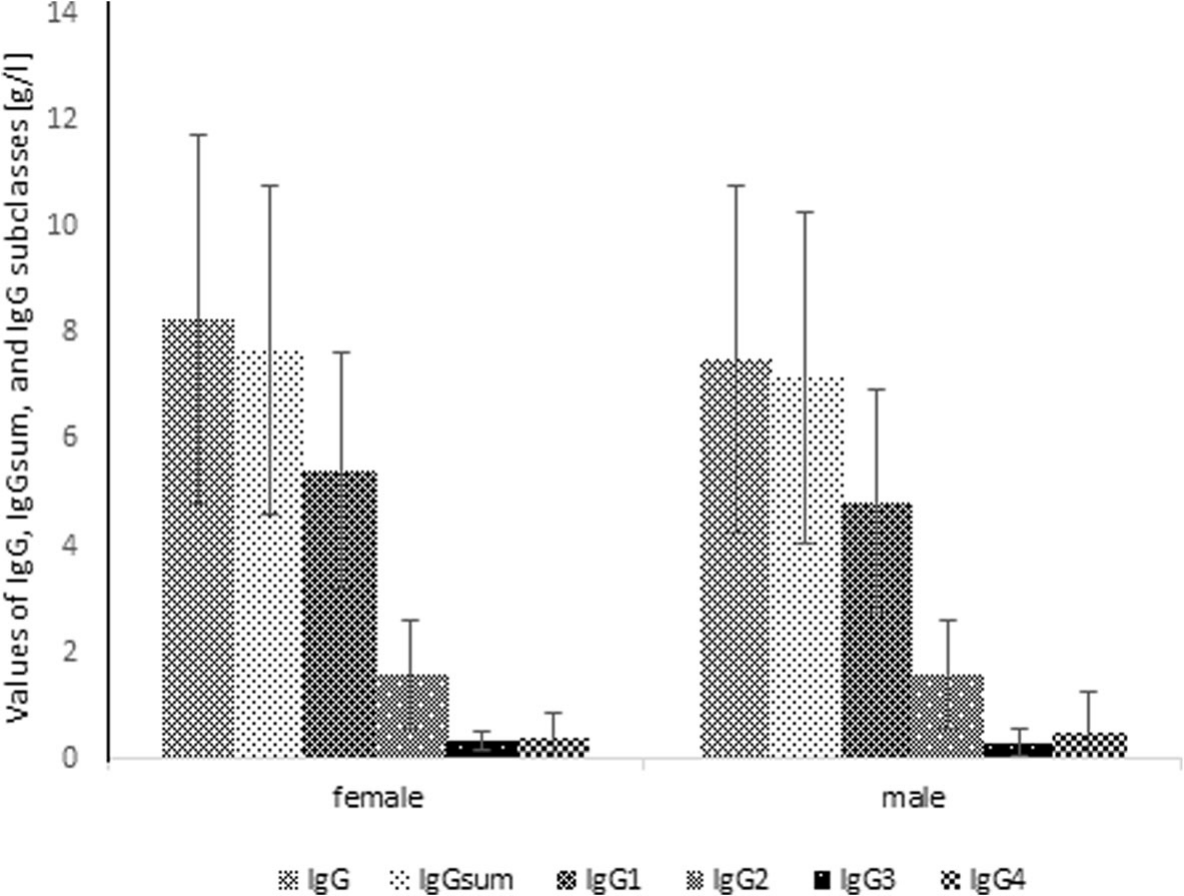
Mean and standard deviation of IgG, IgGsum, and IgG subclasses by gender

Taking into account gender groups, among 245 females patients 198 (80.8%) with D value < 15%, 148 (60.4%) with D value < 10% and 94 (38.4%) with D value < 5% wereobserved. The proportion of subjects with IgG above IgGsum was 74.7, 70.3 and 64.9%, respectively. In the male population, there were 348 (86.4%) patients with D value < 15%, 268 (66.5%) with D value < 10% and 159 (39.5%) with D value < 5%. The proportion of males with IgG higher than IgGsum was 67.5, 62.7 and 54.7%, respectively. In Fig. 2, the percentage of males and females according to D value is presented. The Fig. 3 shows the total concentration of IgG, IgGsum, and IgG subclasses among males and females.

**Fig. 4:**
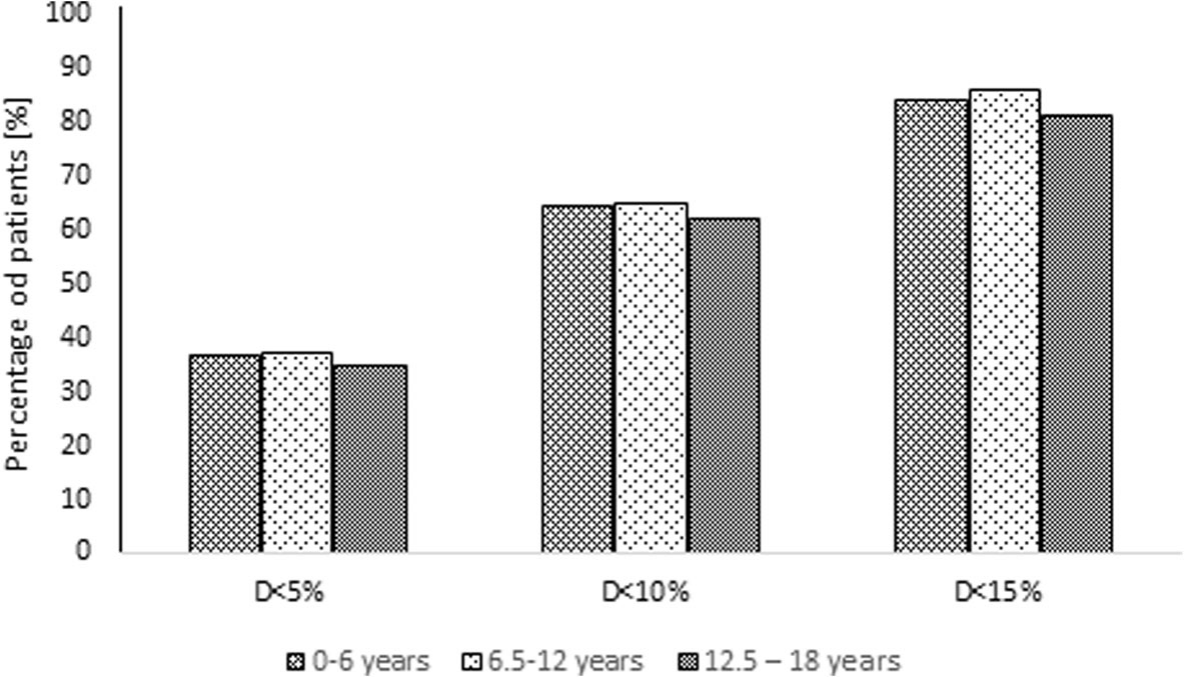
Percentage of patients belonging to a certain age group according to the difference of IgGsum and IgG for the whole patient population

**Fig. 5:**
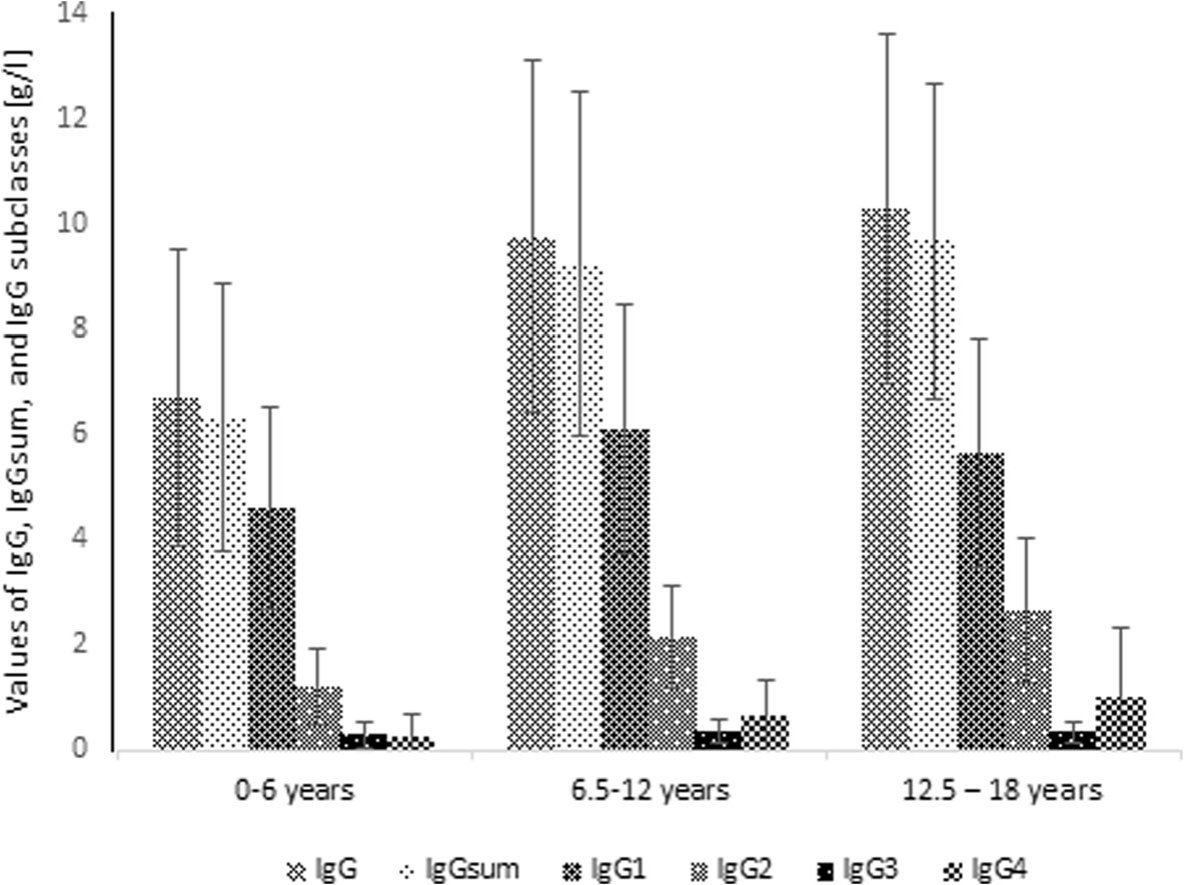
Mean and standard deviation of IgG, IgGsum, and IgG subclasses by age

When considering age groups, among 428 patients aged under 6 years, 360 (84.1%) had D value < 15%, 275 (64.3%) had D value < 10% and 158 (36.9%) had D value < 5%. The proportion of children with IgG greater than IgGsum was 70.6, 65.5 and 58.9%, respectively. In the population of 151 patients aged between 6 and 12 years, there were 130 (86.1%) with D value < 15%, 98 (64.9%) with D value < 10% and 56 (37.1%) with D value < 5%. The proportion of children with IgG higher than IgGsum in this group was 73.1, 69.4 and 58.9%, respectively. Among 69 patients in the age range between 12 and 18 years, 56 (81.2%) with D value < 15%, 43 (62.3%) with D value < 10% and 24 (34.8%) with D value < 5% were observed. The percentage of patients having IgG greater than IgGsum was 60.7, 55.8 and 45.8%, respectively. In Fig. 4, the percentage of patients belonging to a certain age group according to D value is presented. The Fig. 5 shows the total concentration of IgG, IgGsum, and IgG subclasses in certain age group.

**Fig. 6:**
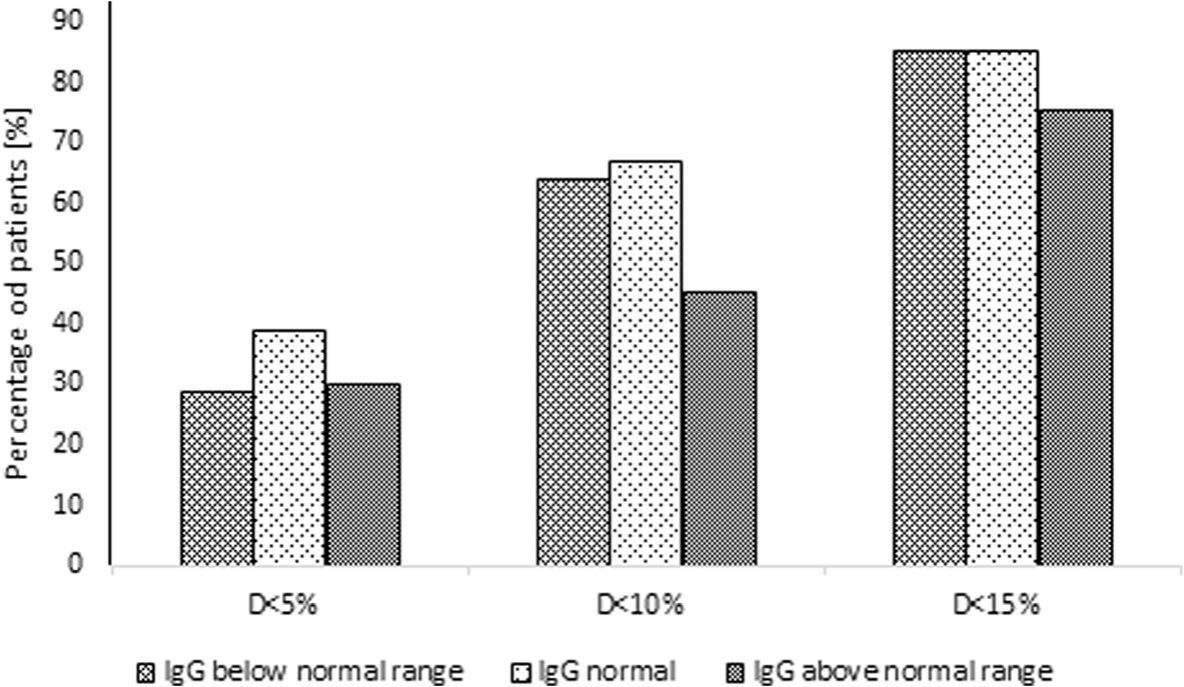
Percentage of patients with certain IgG abnormality according to the difference of IgGsum and IgG for the whole patient population

**Fig. 7:**
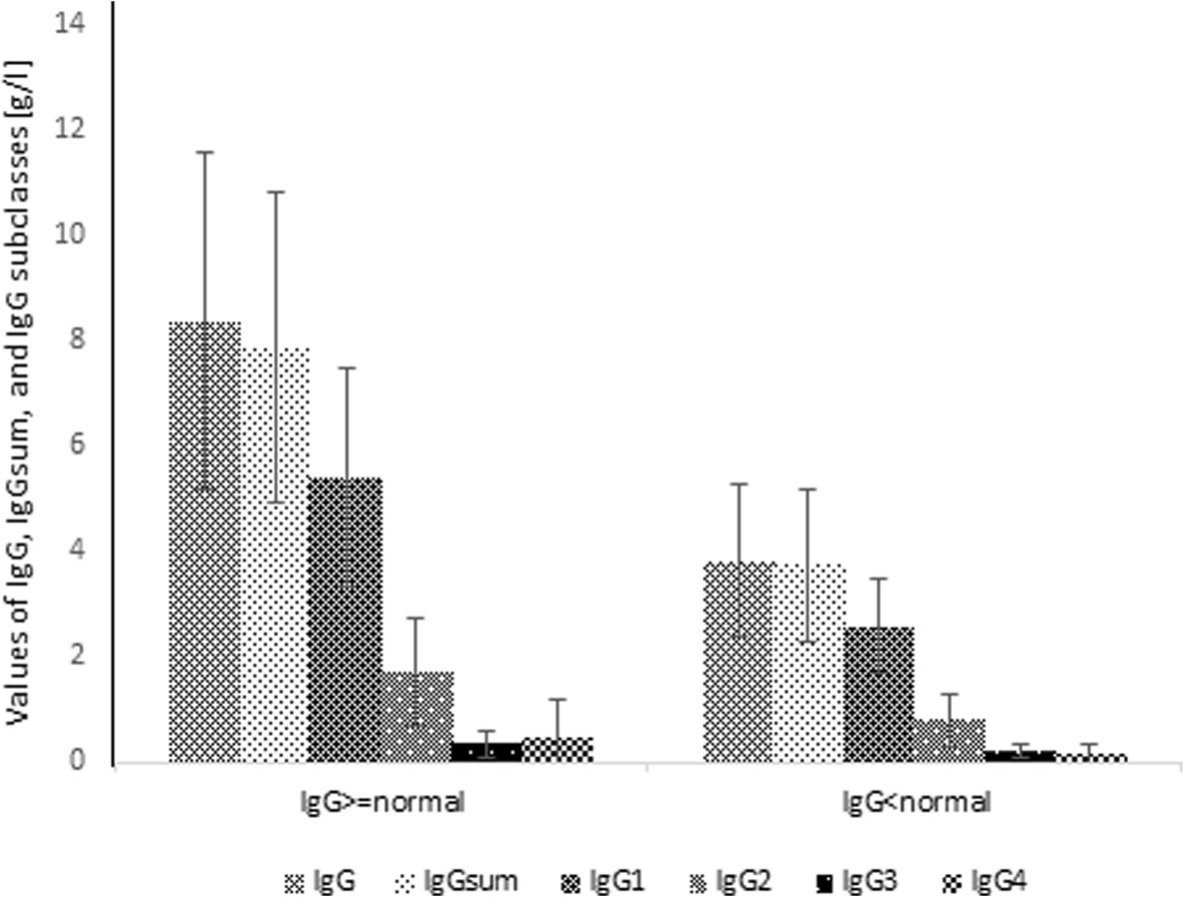
Mean and standard deviation of IgG, IgGsum, and IgG subclasses by IgG abnormality

When analyzing groups according to IgG measured versus a normal IgG range, of80patients with IgG below the normal range, 68 (85.0%) had D value < 15%, 51 (63.8%) had D value < 10% and 23 (28.8%) had D value < 5%. The proportion of patients with IgG greater than IgGsum was 58.8, 54.9 and 56.5%, respectively. Among 496 patients with IgG normal, there were 423 (85.3%) with D value < 15%, 333 (67.1%) with D value < 10% and 193 (38.9%) with D value < 5%. The percentage of children having IgG greater than IgGsum was 70.7, 66.7 and 59.6%, respectively. In the population of 73 patients having IgG above the normal range, 55 (75.3%) with D value < 15%, 33 (45.2%) with D value < 10% and 22 (30.1%) with D value < 5% were observed. The proportion of children with IgG higher than IgGsum in this group was 81.8, 66.7 and 54.5%, respectively. In Fig. 6, the percentage of patients having IgG normal, below or above normal range according to D value is shown. The Fig. 7 shows the total concentration of IgG, IgGsum, and IgG subclasses for patients with immunodeficiency and those with IgG normal and above normal range.

**Fig. 8:**
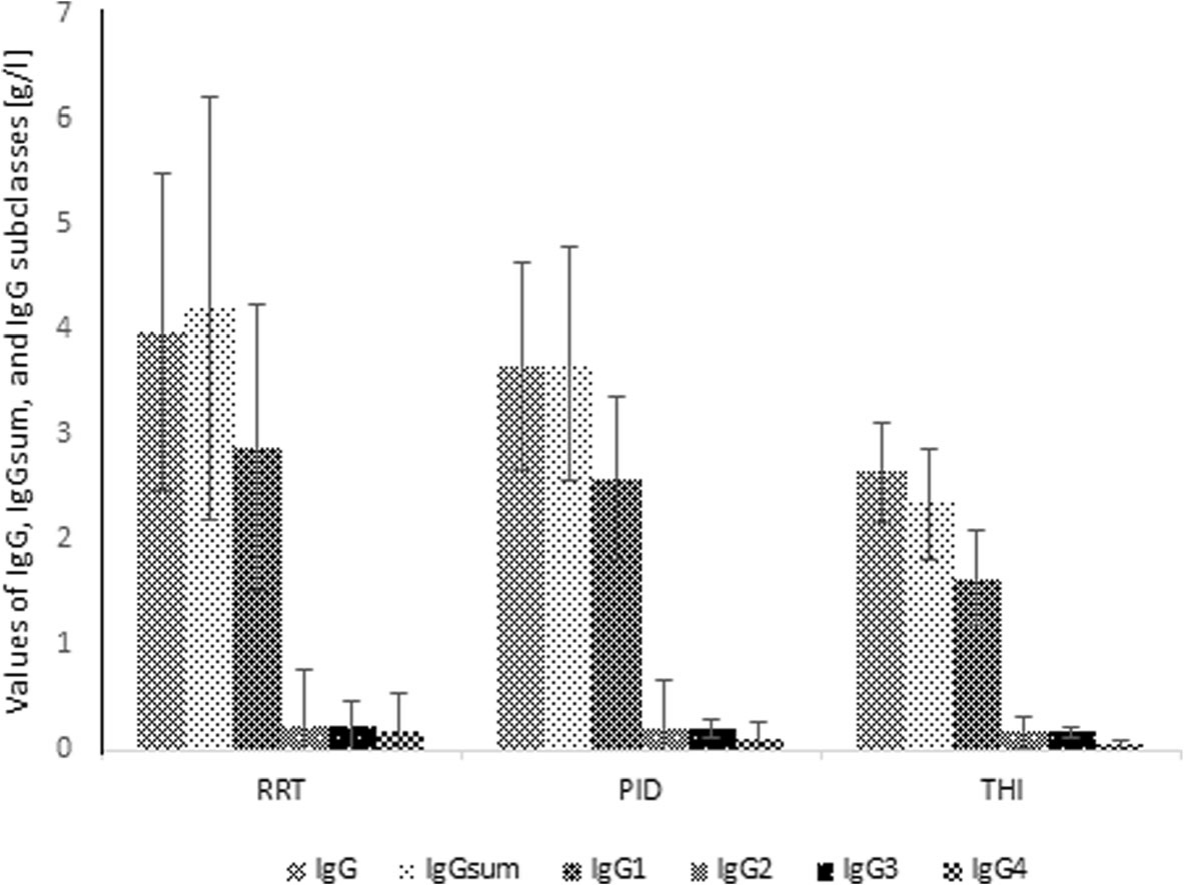
Mean and standard deviation of IgG, IgGsum, and IgG subclasses by clinical diagnosis

The figure below (Fig. 8) shows the results of IgG, IgGsum, and IgG subclasses of patients with total IgG deficiency divided into 3 clinical groups: recurrent respiratory tract infections (RRT), primary immunodeficiency (PID) i transient hypogammaglobulinemia of infancy (THI). The Mann-Whitney U test indicated that there are no significant differences in IgG, IgGsum, and IgG subclasses between clinical diagnosis.In addition, a whole group of patients (670) was analyzed for clinical diagnosis and shortages in subclasses as presented in Table 2.

**Table 2:** The percentage of patients with a deficiency of IgG subclasses division for clinical diagnosis

IgG subclasses	RRT [%]	PID [%]	THI [%]
IgG1	81	15	4
IgG2	76	24	–
IgG3	79	21	–
IgG4	84	16	–

Considering gender, a higher percentage of patients with D value > 15% and a higher percentage of patients with IgG greater than IgGsum is observed in the female group than in the male group. The average value of discrepancy between IgG and IgGsum was recognized more in the female group (9.4%) than in the male group (8.5%). In the case of age groups, a significantly lower percentage of patients with D value > 15% is observed for children aged between 12 and 18 years. The percentage of patients with IgG greater than IgGsum decreases as age increases. The average D value is highest for the group aged between 12 and 18 years (9.3%). Comparable values were recognized for other groups – 8.8% for children aged under 6 years and 8.6% for children aged between 6 and 12 years. Analyzing IgG abnormality groups, it can be concluded that for the population with IgG greater than IgGsum, there is a lower proportion of patients with IgG below normal range than with IgG normal or above normal range. The average values of discrepancy between IgG and IgGsum were: 9.1% for children with IgG below normal range, 8.5% for children with IgG normal and 10.7% for children with IgG above normal range.

### Summary

The study analyzed the effects of three parameters: age, sex, and IgG deviation from normal to the value of the difference between IgG and the sum of IgG of each subclass (IgGsum = IgG1 + IgG2 + IgG3 + IgG4). Patients were divided into groups according to these parameters:

1. women and men
2. age up to 6 years, between 6 and 12 years, and between 12 and 18 years
3. IgG below norm, IgG norm and IgG above norm.

The main purpose of the analysis was to determine whether the values of the aforementioned parameters significantly affects the difference between IgG and IgGsum.

Patients’ total IgG values were on average higher than IgGsum and there is a statistically significant difference (*p* < 0.05) between total IgG and IgGsum. In the results published by McLean-Tooke et al., for a group of adult patients, the tendency was opposite. This shows that there is a difference between children and adults in this respect.

Statistical analysis (Kruskal-Wallis test) indicates that all three parameters: age, sex, and IgG deviation from the norm, have statistically significant effects on the amount of difference between IgG and IgGsum.

In the case of sex, the greater mean difference between IgG and IgGsum was observed in women. In the group of women, we observed a greater proportion of patients with IgG and the difference between IgGsum exceeding 15% of patients, and for which IgG is greater than IgGsum.

In the case of age, the oldest children had significantly fewer patients with a percentage of the difference between the total IgG and IgGsum exceeding 15% than in the other two child age groups. Increase in patient age results in an increase in the difference between IgG and IgGsum and a decrease in the number of patients for whom IgG is greater than IgGsum.

Analyzing the deviation of the total IgG from the norm, we found that the average value difference between IgG and IgGsum is the smallest in patients with normal IgG. Deviation from the normal range (up or down) results in an increase in the mean difference between total IgG and IgGsum.

## Conclusion

Determination of serum immunoglobulin levels is an increasingly available and relatively inexpensive laboratory test performed not only in specialized departments but also in primary care. Assessment of IgG levels as well as IgA and IgM is one of the fundamental studies on the function of the immune system. The indications for their markings are very different and primarily include diagnostics for primary and secondary immunodeficiencies.

In addition, we have shown the reliability of the results obtained. Their determination in two different laboratories and on different analyzers, as well as the freezing process, does not adversely affect the test results.

## Abbreviations

CVID: Common variable immunodeficiency
PID: Primary Immunodeficiency
RRT: Recurrent respiratory tract infections
THI: Transient hypogammaglobulinemia of infancy
